# Upconversion optical entropy encoding for infrared complex-amplitude imaging

**DOI:** 10.1038/s41377-026-02215-7

**Published:** 2026-03-09

**Authors:** Sheng-ke Zhu, Tuqiang Pan, Chao-xian Tang, Ai-Hua Li, Ze-huan Zheng, Yi Xu, Xiangping Li, Jin-hui Chen

**Affiliations:** 1 Institute of Electromagnetics and Acoustics, Key Laboratory of Electromagnetic Wave Science and Detection Technology, Xiamen University, Xiamen, China; 2 Key Laboratory of Photonic Technology for Integrated Sensing and Communication, Ministry of Education of China, Guangdong University of Technology, Guangzhou, China; 3 Institute of Advanced Photonics Technology, School of Information Engineering, Guangdong University of Technology, Guangzhou, China; 4 Department of Physics, Xiamen University, Xiamen, China; 5 Institute of Photonic Technology, College of Physics and Optoelectronic Engineering, Jinan University, Guangzhou, China; 6 Shenzhen Research Institute of Xiamen University, Shenzhen, China; 7 Innovation Laboratory for Sciences and Technologies of Energy Materials of Fujian Province (IKKEM), Xiamen, China

**Keywords:** Imaging and sensing, Photonic devices

## Abstract

Upconversion detection of infrared radiation by cost-effective silicon photodetectors in visible bands has spurred a revolution in infrared imaging technology, unlocking a wide range of applications in biological imaging, optical spectroscopy, and optical data storage. Despite significant progress in upconversion detection, real-time, concurrent, complex-amplitude imaging of both phase and amplitude information, indispensable for disclosing the full signature of infrared scenes, remains a daunting challenge, impeding their widespread applications. By integrating the unique advantages of both coherent and incoherent approaches, we propose the concept of upconversion optical entropy encoding and demonstrate a video-rate infrared complex-amplitude imaging system. This is achieved by leveraging the synergistic interaction between light scattering in disordered photonic structures and lanthanide upconversion photoluminescence. By tailoring the information entropy of upconversion speckles, infrared light-field information can be captured in a single visible snapshot and explicitly reconstructed, assisted by a deep learning network, enabling infrared complex-amplitude imaging at a video rate of 25 frames per second (fps) and with high-fidelity 8-bit grayscale modulation. The high photosensitivity of the developed infrared imaging system enables a power detection limit of 0.2 nW μm^−2^, three orders of magnitude lower than that of conventional parametric upconversion imaging. As a proof of concept, we demonstrate its applications in capturing video frames of natural scene images and classifying images of speed-limit signs for autonomous driving. This approach can be readily integrated with other cross-band imaging methods, paving the way for various infrared application scenarios that require video-rate, high-photosensitivity, and high-fidelity protocols.

## Abstract (web summary)

We demonstrate upconversion optical entropy encoding for infrared complex-amplitude imaging by leveraging the synergistic interaction between light scattering in disordered photonic structures and lanthanide upconversion photoluminescence.

## Graphical abstract

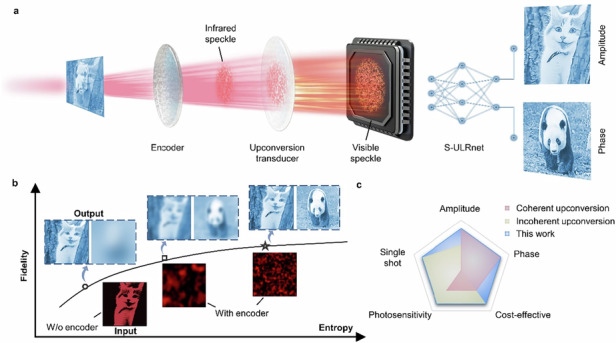

## Introduction

**Fig. 1:**
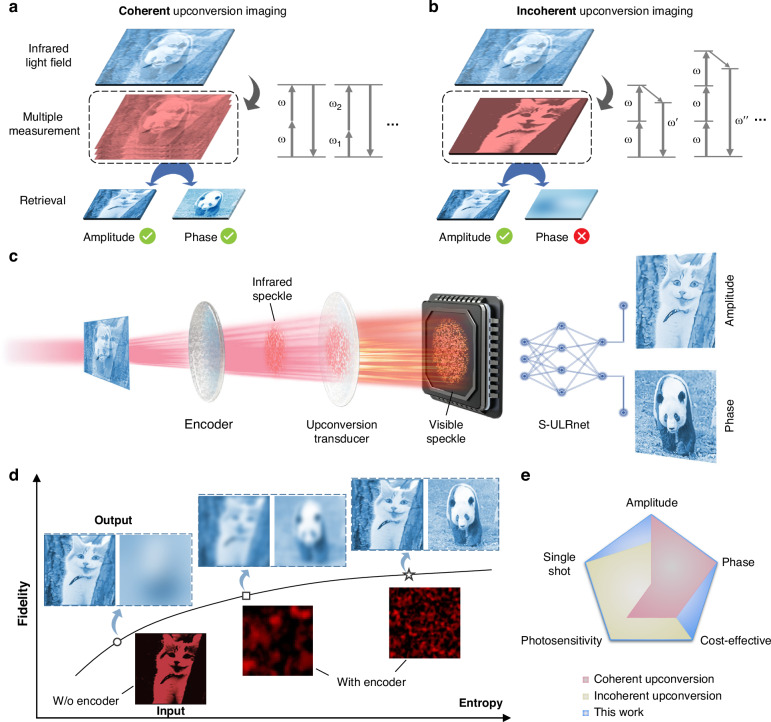
Concept of complex-amplitude infrared light field imaging using upconversion optical entropy encoding. **a** Illustration of coherent upconversion imaging of infrared light via parametric oscillation. The right panel shows the typical parametric processes of second-harmonic generation, sum frequency generation, etc. These coherent nonlinear processes, generated by nonlinear crystals or metasurfaces, often require high pump power. The amplitude and phase information of incident light are reserved during coherent upconversion and can thus be retrieved in principle. **b** The incoherent upconversion imaging via multiphoton luminescence process. The right panel shows the typical two-photon and three-photon luminescence generated by lanthanide transducers. The phase information of incident light is lost in the excited luminescence. **c** Schematic of the intelligent infrared light-field imaging system composed of the entropy encoder, upconversion transducer, and S-ULRnet. Light amplitude and phase information are encoded in the upconversion speckle patterns, which can be retrieved with the assistance of S-ULRnet. **d** The dependence of retrieval fidelity of light-field images on the entropy of optical encoder. The phase information cannot be retrieved without the disordered encoder. With an optical encoder, imaging fidelity improves as entropy increases. The insets are schematic illustrations of retrieved amplitude and phase images. The illustrated images are adapted from ImageNet^54^. **e** Performance comparisons of infrared-to-visible upconversion imaging devices among existing upconversion imaging platforms

Infrared detection and imaging technologies are actively pursued due to their crucial roles in fields of molecular fingerprint spectroscopy^1–5^, optical data storage^6,7^, biological imaging^8–12^, night vision^13^, light detection and ranging^14,15^, to name a few. However, most commercial infrared image sensors rely heavily on costly epitaxial semiconductors operating at cryogenic temperatures, which prevents their widespread use. Recently, frequency upconversion detection, in which low-energy infrared radiation is sophisticatedly translated into visible bands for static detection using cost-effective silicon-based image sensors (SIS), has emerged as a promising complementary solution^16–28^. At present, two primary approaches have been exploited for infrared upconversion imaging: coherent upconversion via parametric oscillations and incoherent upconversion utilizing multiphoton luminescence, as illustrated in Fig. 1a, b. Coherent upconversion imaging can be achieved using bulk nonlinear crystals or compact metasurfaces^18,20,29–31^, which inherently require high pump power and pose a challenge for practical applications. Moreover, the delicate polarization control and precise phase-matching conditions demanded to formulate efficient nonlinear conversions, such as four-wave mixing and harmonic generation^18,32–34^, prevent it from retrieving complex-amplitude information of the light field in a simple and real-time manner^29,32,35,36^. Alternatively, lanthanide-based upconversion luminescence offers another promising approach for infrared imaging^8,16,17^. Unlike coherent upconversion, incoherent upconversion of lanthanide transducers is generated by the sequential absorption of two or more infrared photons, producing visible-band emissions with significantly lower threshold power^16,19,37^. Nevertheless, the incoherent nature of multiphoton luminescence inherently omits the phase, one of the most important signatures of the light field, thus hindering the holistic light-field retrieval even with bulky optical interference systems. Although these endeavors push the state-of-the-art capabilities of upconversion imaging, achieving a high frame rate and high-fidelity light-field detection of both amplitude and phase information, which are essential for revealing the full characteristics of infrared scenes, remains a daunting challenge.

In this study, we report a video-rate, high-photosensitivity, and high-fidelity short-wave-infrared (SWIR) light field imaging system utilizing upconversion optical entropy encoding, as illustrated in Fig. 1c. This upconversion encoding method leverages multiple light scattering by cascading an optical encoder and a lanthanide transducer to map complex-amplitude SWIR light comprising both amplitude and phase information into single snapshots of visible-intensity speckles, which are then acquired by an SIS. To reconstruct the light field, we introduce the speckle-upconversion light field retrieval network (S-ULRnet), a deep neural network (DNN) capable of real-time retrieving the SWIR light field from its corresponding upconverted snapshots. Importantly, we reveal that the information entropy of the upconversion speckles, tailored by the optical encoder, plays a crucial role in optimizing the performance of the upconversion imaging system (UIS), as shown in Fig. 1d. The demonstrated UIS achieves exceptional performance, including a high frame rate of up to 25 fps, low-power detection limit down to 0.2 nW μm^−2^ (@1550 nm), and high-fidelity imaging of 8-bit grayscale modulation of both amplitude and phase, outperforming existing upconversion infrared imaging approaches (see comparisons in Fig. 1e and Supplementary Note 1). As a proof of concept, we demonstrate the potential applications of such UIS for capturing videos of natural scene images and speed-limit signs for autonomous driving with real-time capability. This intelligent upconversion imaging approach is adaptable to various infrared imaging systems, offering an integrated, cost-effective protocol for high-dimensional light-field detection with fast speed and high fidelity.

## Results

### Principles of upconversion optical encoding for complex-amplitude infrared imaging

**Fig. 2:**
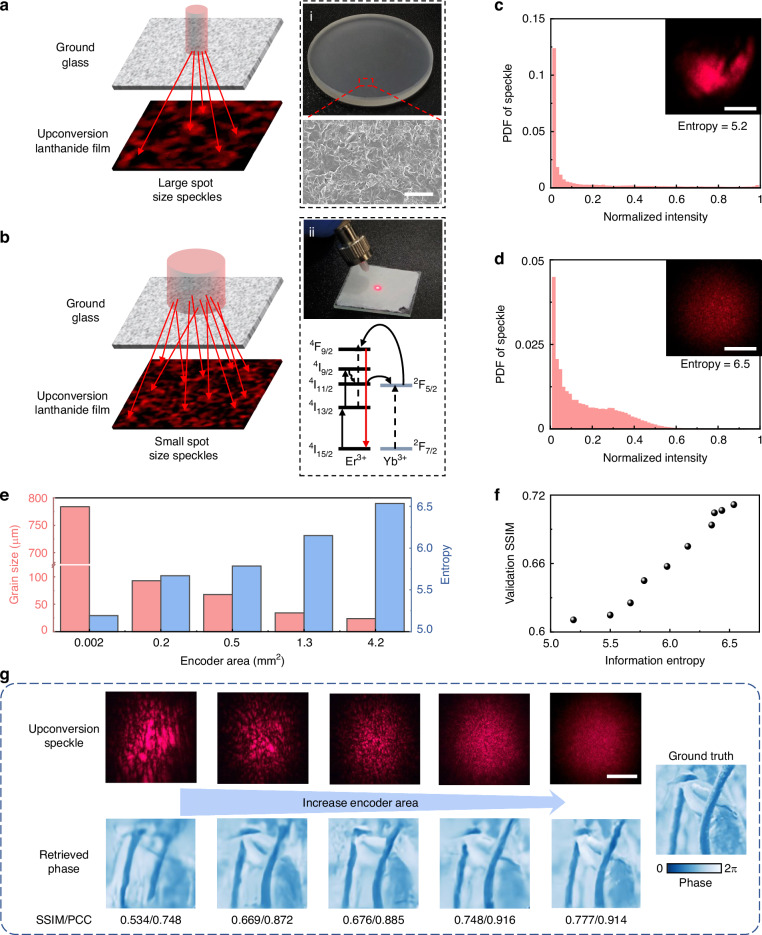
Tailoring the information entropy for short-wave-infrared (SWIR) upconversion imaging. The grain size of speckles produced by a ground glass under the illumination of SWIR light using a small beam waist (**a**) and a large beam waist (**b**). The panels: (i) Photograph of the ground glass (optical encoder) with a diameter of 1 inch, where the enlarged view shows the scanning electron microscope image of the ground glass. The scale bar is 20 μm; (ii) The photograph of upconversion lanthanide film emitting red light (@660 nm) when illuminated by SWIR light (@1550 nm). The lower panel shows the upconversion fluorescence mechanism of the lanthanide transducer based on the energy transfer between Er^3+^ and Yb^3+^ ions (see details in Supplementary Note 2). Measured probability density function (PDF) for upconversion speckle intensity with different information entropy: (**c**) entropy of 5.2, (**d**) entropy of 6.5. The insets show the corresponding speckle patterns. The scale bar is 1 mm. **e** Dependence of grain size and the information entropy of upconversion speckle on the encoder area. **f** Dependence of phase retrieval structural similarity index (SSIM) on the entropy of upconversion speckle. The validation SSIM is averaged over 5000 measurements. **g** Single-shot upconversion speckles and their corresponding retrieved phase images. The speckle grain size is tailored by adjusting the encoder area. The SSIM and Pearson correlation coefficient (PCC) values of retrieved phase images are presented. The ground truth is also provided for comparison. The scale bar is 1 mm

To acquire light field information via a single-shot measurement of visible speckles, the incident SWIR light is encoded by a ground glass that serves as a photonic encoder with disordered hierarchical microstructures (right panel of Fig. 2a). This optical encoder maps the input SWIR light comprising amplitude and phase information to a speckle-like optical pattern through multiple linear scatterings^38^. A lanthanide transducer film cascaded with a ground glass efficiently converts the scattering SWIR photons into visible photons, which can be detected by an SIS^16,17^ (right panel of Fig. 2b, Supplementary Note 2). The upconversion luminescence excited by the encoded SWIR speckle fields carries implicit light-field information during detection. The complicated nonlinear mapping relationship between the input complex-amplitude SWIR light and the resulting incoherent upconversion speckles can be formally expressed as follows (see Supplementary Note 3 for more details):

1 $$I({\boldsymbol{r}})=F({{|T}{\rm{\cdot }}{{\boldsymbol{E}}}_{{in}}({\boldsymbol{r}})|}^{2})=H({{\boldsymbol{E}}}_{{in}}({\boldsymbol{r}}))$$

where ${{\boldsymbol{E}}}_{{in}}({\boldsymbol{r}})$ is the input SWIR light field; $T$ is the transmission matrix of the ground glass that relates ${{\boldsymbol{E}}}_{{in}}({\boldsymbol{r}})$ to the SWIR speckle field^39,40^; $F(\cdot)$ denotes the upconversion process from the SWIR speckles to the visible speckles $I({\boldsymbol{r}})$ based on the complicated multiphoton luminescence of the lanthanide ions (Er^3+^-Yb^3+^)^41,42^, and $H(\cdot)$ represents the mapping relationship between the input SWIR light field ${{\boldsymbol{E}}}_{{in}}$ and the intensity distribution of the visible speckles $I({\boldsymbol{r}})$. Note that ${{\boldsymbol{E}}}_{{in}}$ is a complex number containing both spatial amplitude and phase information:

2 $${{\boldsymbol{E}}}_{{in}}=\hat{e}{\rm{\cdot }}A({\boldsymbol{r}}){e}^{i\phi ({\boldsymbol{r}})}$$

where $A({\boldsymbol{r}})$ and $\phi ({\boldsymbol{r}})$ correspond to the amplitude and phase information of the incident light field, respectively, and $\hat{e}$ denotes the unit vector of the electric field. Equations (1–2) show that the optical encoder enables the encoding of phase information within the SWIR speckle intensity ${{|T\cdot }{E}_{{in}}({\boldsymbol{r}})|}^{2}$, where a light field composed of intricate amplitude and phase information can lead to diverse speckle patterns with distinct spatial distributions, as shown in Fig. 1d. Currently, no analytical formula can explicitly describe the relationship between the upconversion speckle intensity and input SWIR light field for the complicated physical processes of multiple optical scatterings and incoherent upconversion light-matter interactions. Consequently, the inverse mapping function ${H}^{-1}$ that relates the upconversion speckle intensity $I$ to the input SWIR light field ${{\boldsymbol{E}}}_{{in}}$ (${{\boldsymbol{E}}}_{{in}}$ = ${H}^{-1}(I)$) cannot be derived explicitly (see Supplementary Note 3 for details). Herein, the inverse mapping function ${H}^{-1}$ is retrieved using a data-driven deep learning approach^43–48^. DNNs are adept at capturing complicated mapping relationships in diverse physical systems and exhibit robustness against noise in measurement systems^35,40,41,43–47,49–51^. To retrieve the complex-amplitude SWIR light field (${{\boldsymbol{E}}}_{{in}}$) from a single-shot intensity measurement, the S-ULRnet is developed and trained to learn the complicated mapping relation ${H}^{-1}$.

In this case, the speckle pattern should possess sufficient information, which necessitates generating a large number of scattering channels within the optical encoder. Specifically, the number of scattering channels in the ground glass is fundamentally related to the effective size of the incident and detected apertures^39^. In practical applications, the detected aperture size remains fixed, allowing the effective incident-aperture size to be adjusted to manipulate multiple-scattering properties, thereby optimizing the fidelity of the intelligent UIS. This effective aperture size can be controlled by varying the waist of the illuminating light beam, as illustrated in Fig. 2a and b. To quantify this effect, we calculate the information entropy derived from the probability density function (PDF) of the speckle intensity distributions (see Supplementary Note 4)^52^. The statistical PDF of the upconversion speckle intensity exhibits a distinct dependence on the waist size of the illuminating light beam, which transitions from concentrated states (Fig. 2c) to broader diffusion distributions (Fig. 2d). This shift is characterized by a substantial reduction in the size of the speckle grain. By increasing the width of the beam waist, the speckle grain size progressively decreases, increasing the information entropy of the speckle image (Fig. 2e). Changes in speckle entropy are manifested as variations in the spatial frequency content of the patterns and directly indicate the information capacity of the speckle image. As the S-ULRnet is trained in an end-to-end manner, the information entropy of the upconversion speckle image correlates with the performance of S-ULRnet. This relationship is experimentally validated in Fig. 2f, which shows the dependence of retrieval fidelity, as measured by the structural similarity index (SSIM), on speckle entropy. Higher information entropy implies more information transmission channels, thereby improving upconversion imaging performance and fidelity. Figure 2g presents the visual phase retrieval results obtained by increasing the encoder area, where the corresponding upconversion speckles are also shown. The positive correlation between retrieval fidelity and speckle entropy underscores the critical role of speckle entropy in enhancing imaging accuracy.

### Complex-amplitude infrared upconversion imaging performance

**Fig. 3:**
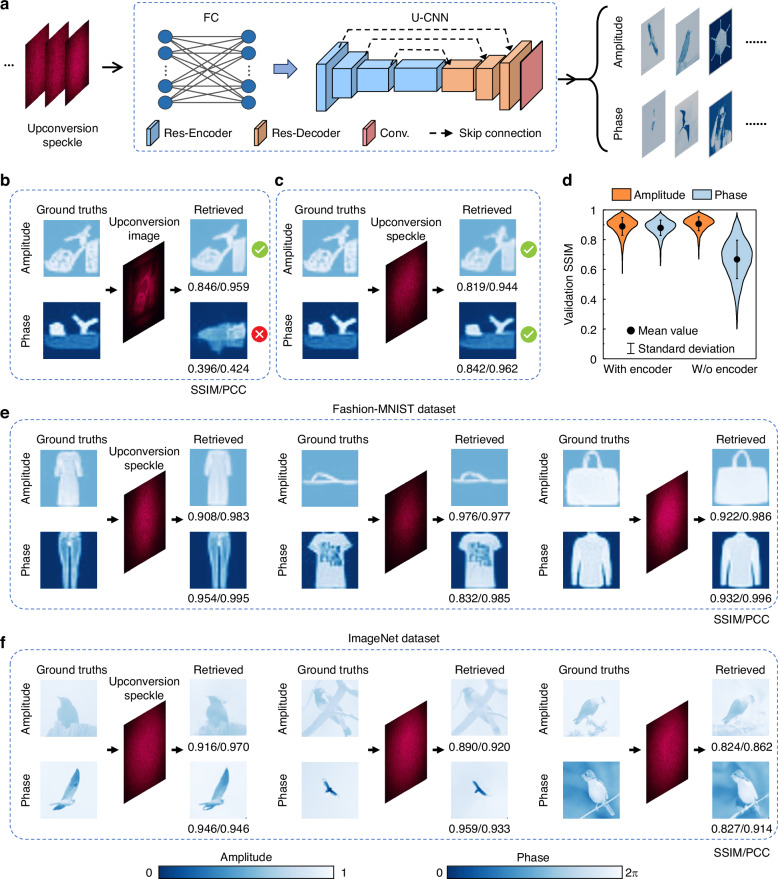
Upconversion imaging performance of SWIR light field. **a** The schematic illustration of S-ULRnet. The architecture of the S-ULRnet includes a fully connected (FC) block, four Res-Encoders with downsampling, three Res-Decoders, and one output convolutional layer (Conv) for channel compressing with upsampling. Skip connections are established among the first three down-sampling and the up-sampling modules. **b** Conventional upconversion imaging without an encoder fails to retrieve phase information. **c** The proposed upconversion imaging with an encoder for complex-amplitude light field retrieval. **d** Violin plots of the distributions of validation SSIM of the reconstructed complex-amplitude image, with and without the encoder. Each violin’s width reflects the distribution of the validation SSIM values and its probability density. Within each violin, the black dot represents the mean value; the error bar indicates the standard deviation. The statistical results are based on 3000 measurements from the Fashion-MNIST dataset^61^. The ground truths, the single-shot upconversion speckle, and the corresponding retrieved light-field information by the S-ULRnet are shown using the images adapted from (**e**) Fashion-MNIST dataset and (**f**) ImageNet dataset^54^

The light field retrieval is assisted by the specially-designed S-ULRnet composed of a fully connected layer (FC) and a U-shaped convolutional neural network (U-CNN) to output both amplitude and phase information (Fig. 3a; see Methods, Supplementary Note 5). Adding the FC layer can result in a substantial reduction in both training and validation loss, confirming its necessity for boosting the model’s performance (see Supplementary Note 5). The complex-amplitude SWIR light field is generated using a commercial spatial light modulator-based optical system (see Methods and Supplementary Note 6). In a conventional upconversion imaging framework, when the complex-amplitude light field is upconverted by the lanthanide transducers without pre-encoding and subsequently captured by a visible camera, only the amplitude information can be recovered from the single-shot speckle, as shown in Fig. 3b. Despite the inherent noise in the upconversion process, the S-ULRnet demonstrates robustness in decoding the input amplitude-based image with high fidelity. When an optical encoder is incorporated, the S-ULRnet successfully decodes both the amplitude and phase information from the single-shot upconversion speckle simultaneously, as illustrated in Fig. 3c. In Fig. 3d, the violin plots illustrate the fidelity distribution of amplitude and phase reconstruction results for upconversion imaging with and without the optical encoder. A notable observation from the results is that the optical encoder significantly improves the average fidelity of phase retrieval and reduces its standard deviation, underscoring the critical role of optical encoding in achieving high-accuracy light-field reconstruction.

Figure 3e shows typical retrieval results of the SWIR complex-amplitude light field based on single-shot upconversion speckles, with images adapted from the Fashion-MNIST dataset. The retrieved fidelity, quantified by the SSIM and the Pearson correlation coefficient (PCC), is indicated for each image. Both the amplitude and phase information encoded in upconversion speckles are retrieved with high fidelity. The average SSIM/PCC of the retrieved complex-amplitude images over 3000 validations is as high as 0.884/0.974. Notably, compared to previously reported upconversion imaging works^19,20,29,31,32^, the proposed method achieves a high dynamic range of modulation, with an 8-bit grayscale for both the amplitude and phase (see Supplementary Note 7). At the same time, the developed UIS shows high infrared photosensitivity, with a power detection limit of 0.2 nW μm^−2^ (see Methods and Supplementary Note 7), which is more than three orders of magnitude lower than that of upconversion metasurfaces and nonlinear bulk crystals (see Supplementary Note 1). The photosensitivity of the developed UIS can be further optimized by engineering the lanthanide transducer’s rare-earth doping^53^. Such high photosensitivity enables video-rate imaging at 25 fps (see Supplementary Note 6 for detailed time consumption of the imaging process), where each frame requires an exposure time of 35 ms and an S-ULRnet inference time of 5 ms. To our knowledge, this is the first demonstration of complex-amplitude upconversion imaging in a single-shot manner. To further assess the performance and generalizability of S-ULRnet, we test more complex information adapted from ImageNet^54^, as shown by the typical results on general natural scene images in Fig. 3f. The average SSIM/PCC of the decoded results, based on 5000 validation measurements, is 0.674/0.741. These visualization results of the retrieved images, along with the average SSIM/PCC values, explicitly demonstrate that the proposed UIS can reconstruct the amplitude and phase information of the input SWIR light field with high fidelity across diverse image types (see additional results in Supplementary Note 8).

### Application of an infrared upconversion imaging system

**Fig. 4:**
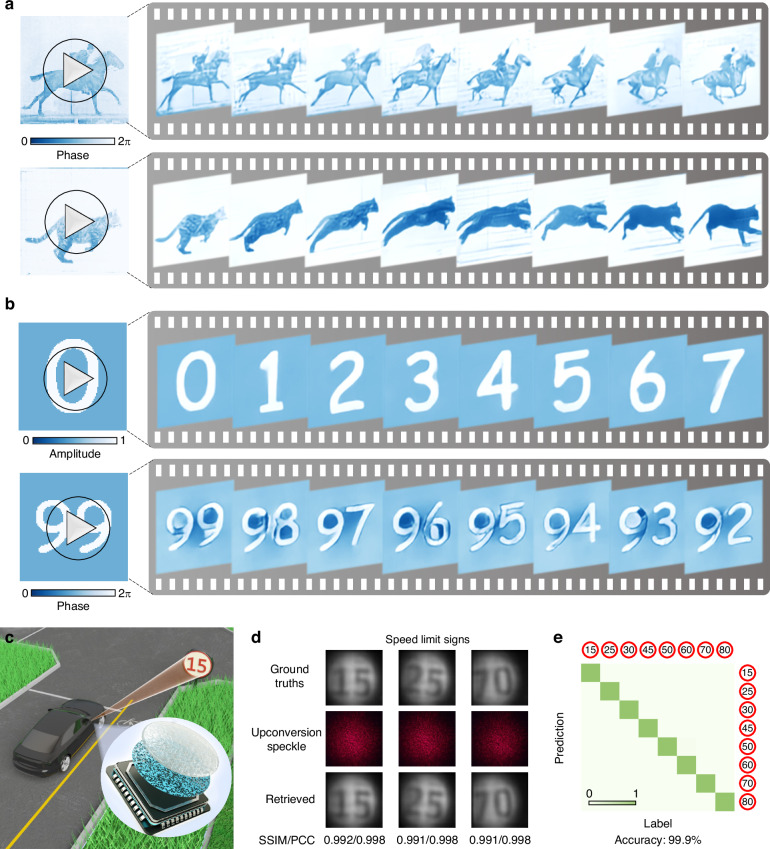
Application of the SWIR upconversion imaging with real-time ability. **a** Experimental demonstration of real-time construction of “horse in motion” (upper panel) and “cat in motion” (lower panel) using the intelligent upconversion imaging technique. The snapshots of Muybridge's recordings from the 1870s marked the historically important breakthrough of the first-ever high-speed photography images. **b** Experimental retrieved results of real-time complex-amplitude imaging for number sequences. Note that these images (**a**, **b**) do not belong to the ImageNet database used for the training of S-ULRnet. **c** Schematic of the upconversion imaging and recognition of traffic speed signs for autonomous driving. **d** Retrieved results of speed-limit sign images. The ground truths, the single-shot upconversion speckle, and the corresponding retrieved amplitude information by the S-ULRnet are shown. **e** The results of classifying speed-limit signs show an accuracy of 99.9%. The used images (ground truths) in (**d**) and (**e**) are adapted from Ref. ^62^

The single-shot imaging capability of the proposed UIS, in combination with the lightweight and high-performance of S-ULRnet, enables the practical imaging applications in natural scenes (Fig. 4a), biological organelles, and irregular patterns (see Supplementary Note 9). As a proof of concept, the real-time application to capture continuous infrared complex-amplitude light-field video (25 fps) is shown in Fig. 4b (see Supplementary Video 1). We consider snapshots in numbers as continuous-frame inputs, which do not belong to the ImageNet database used for training and are not used in the training process. These experimental results demonstrate the superior generalization ability of S-ULRnet, validating that the proposed UIS can effectively handle SWIR light fields in various scenarios that differ significantly from those in the training datasets.

Moreover, we demonstrate the retrieval and classification of speed-limit sign images for autonomous driving using the developed UIS (Fig. 4c). The retrieved results show that amplitude-encoded speed-limit signs can be captured and decoded with high fidelity (Fig. 4d; see additional results in Supplementary Note 9). The average SSIM/PCC of the retrieved images is 0.932/0.954. Figure 4e shows the confusion matrix of classifying speed-limit sign images based on the captured upconversion speckles processed by Resnet50^55^ (see details in Supplementary Note 10), with a classification accuracy of 99.9%. These results conclusively demonstrate that the proposed intelligent infrared image sensor achieves video-rate, high-fidelity light-field imaging, highlighting its potential for cost-effective SWIR video detection technologies.

## Discussion

We demonstrate a video-rate, high-photosensitivity, and high-fidelity infrared light-field imaging system. This strategy is enabled by an upconversion optical encoding approach that implicitly encodes the complete wavefront, comprising both amplitude and phase information, in speckle patterns. Lanthanide upconversion transducers can convert infrared radiation into visible light for acquisition by an SIS in a cost-effective, single-shot fashion. Subsequently, the light fields can be fully retrieved with a high-performance neural network, achieving video-rate speed and photosensitivity with a power detection limit down to 0.2 nW μm^−2^ and a high-fidelity of 8-bit grayscale. Compared to the classical coherent frequency upconversion scheme, our method addresses the challenges of high laser power consumption and complex measurements, enabling video-rate infrared light-field imaging. Compared with current snapshot approaches, our method achieves superior high-fidelity performance, providing complete wavefront information and full dynamic range. Although we primarily focus on results for a specific spectral band of SWIR (@1550 nm), lanthanide transducers with rich energy levels allow multi-band light absorption, facilitating the upconversion of multi-wavelength SWIR light into visible light^16,41,42^. This multi-band absorption can expand the working bandwidth of the proposed upconversion imaging system. For example, benefiting from the energy levels of Yb^3+^ ion (see Supplementary Note 11), the employed lanthanide transducer film can also emit upconversion visible light under 980 nm light excitation. Consequently, this approach can be generalized to other infrared bands. We also performed upconversion imaging using a broadband infrared light source, achieving reasonably good retrieval accuracy, which is crucial for practical applications (see Supplementary Note 11). Notably, the stability of the proposed imaging system is excellent (see Supplementary Note 12), highlighting the robustness of this intelligent SWIR light-field imaging approach. Upconversion nanoparticles (UCNPs) represent an important alternative for infrared light conversion, particularly given their advantages in nanoscale dispersion and biocompatibility^16^. Using UCNPs for upconversion light field imaging can be an exciting avenue for our future research.

All these results consolidate the exceptional ability of the proposed intelligent UIS to retrieve the complex amplitude of the light field across a broad spectral range compared with the narrowband operations of parametric oscillations via nonlinear crystals^18,32,33,56^. Our approach can be generalized to multidimensional infrared imaging techniques, such as hyperspectral imaging and polarization imaging^4,24,57^. The demonstration of the video-rate SWIR light-field upconversion imaging provides a new paradigm in cross-band imaging, which holds great potential in diversified fields such as biomedical imaging, food and agriculture, materials science, and far beyond.

## Materials and methods

### Sample fabrication

The main component of the upconversion phosphor is ZnF_2_(H_2_O)_4_:Yb^3+^/Er^3+^ (Zhanwanglong Technology, Shenzhen, China). Firstly, the upconversion phosphor is added to a test tube containing an appropriate amount of ethanol. The tube is then sealed and placed in an ultrasonic shaker for 30 minutes in a water bath to ensure the upconversion phosphors and ethanol are well mixed. Secondly, the mixed solution is poured onto a clean glass substrate, a glass sheet is used to spread the solution, and the resulting mixture is placed on the spin coater operating at 1000 rpm for 15 s. Finally, the glass substrate is placed on an 80 °C heating table and baked for 2 min to obtain the upconversion transducer film. The film thickness is approximately 100 μm.

### Experimental setup

For the experimental characterizations (see Supplementary Note 6 for more details on the experimental setup), a tunable narrow-linewidth laser source (CTL 1550, Toptica) amplified by an erbium-doped fiber amplifier (EDFA, KG-EDFA-HP-30D-FA) is used as the light source. The laser output is collimated and pre-polarized with horizontal polarization (OPPN05M-NIR1, JCOPTIX, China). The laser beam is subsequently expanded by a combination of a concave lens (L_1_) and a convex lens (L_2_). To generate the target complex-amplitude field $E\left({\boldsymbol{r}}\right)=M\left({\boldsymbol{r}}\right)\exp (i\varPhi ({\boldsymbol{r}}))$ using a phase-only spatial light modulator (SLM, HDSLM80R Plus-TEC, UPOlabs, China), we utilized the established technique detailed in Ref. ^58^. This method encodes the full complex information into a single phase-only pattern. Specifically, the amplitude $M\left({\boldsymbol{r}}\right)$ is encoded by modulating the local diffraction efficiency of a phase grating, while the phase $\varPhi ({\boldsymbol{r}})$ is encoded into the grating’s phase structure. As demonstrated in Ref. ^58^, the first diffraction order of the light modulated by this pattern produces a field whose complex amplitude is directly proportional to the desired target field $E\left({\boldsymbol{r}}\right)$. The first order is spatially filtered to obtain the required complex-amplitude light field for our experiments. The amplitude- and phase-encoded beam is coupled into the scattering medium (ground glass), and the scattered SWIR speckle is further converted to visible light by the lanthanide transducer film. The SWIR light beam waist is scaled using an objective lens (Obj_1_). The upconversion speckle is recorded by an optical imaging system comprising an objective lens, a short-pass filter (OFE1SP-750, JCOPTIX, China), and a camera (MIchrome 5 Pro, Shanghai Taizi Technology). Upconversion detection based on lanthanide material in the experiment operates without any temperature control. A home-built photoluminescence detection system is used to investigate the power response of the prepared upconversion film to 1550 nm laser radiation. To characterize the infrared response of UIS, the integration time of a visible camera is 35 ms. The power detection limit is calculated using the equation: power detection limit = (Minimum detection power)/(effective area), where the minimum detection power is recorded when the camera’s exposure time reaches 35 ms, which is used to achieve the video frame rate. Improving luminescent efficiency through materials engineering^59^ or leveraging optical engineering techniques to compensate for material limitations can further reduce the exposure time. To monitor the time-domain stability of the fluorescence properties of the upconversion material, the PCC, which evaluates the correlation between an instantaneous speckle pattern (every 15 minutes) and the first pattern, is calculated (see Supplementary Note 12).

### Network implementation and computational resource

Figure 3a illustrates the architecture of the S-ULRnet and the information flow of the task (see Supplementary Note 5 for details of the S-ULRnet). The as-captured visible upconversion speckle images are inputs of the S-ULRnet, which outputs the retrieved SWIR light field (amplitude and phase). The S-ULRnet comprises an FC and a U-CNN. The FC layer mimics the physical process of multiple scattering in the ground glass. This layer allows the network to focus more on global information, which aligns with the multiple-scattering effect of the ground glass. The U-CNN is an encoder-decoder structure comprising multiple residual encoders (Res-Encoder) and decoders (Res-Decoder) (see Supplementary Note 5 for details). The Res-Encoder output passes through a skip connection to the Res-Decoder, following the standard Unet configuration. The U-CNN performs channel matching via a convolutional layer, and the channel number *N* = 1 (*N* = 2) represents amplitude/phase-only retrieval (complex-amplitude retrieval).

The parameters of all the used datasets and their corresponding average fidelity are summarized in Supplementary Table 4. For all of the upconversion speckle images, the red channel data of the color images is used as the network input. Each dataset is divided into a training set (90%) and a validation set (10%). The validation dataset is uniformly sampled. The resolution of the speckle output fed to the network is 200 × 200. For complex amplitude image reconstruction, all the images encoded in the amplitude dimension are normalized and scaled from 0–1 to 0.5–1. All neural network models are implemented using Python 3.9.13 in PyTorch 2.0.0. The workstation used to train and test the neural network is equipped with an Nvidia RTX4090 graphics card.

### Manipulating the entropy of the upconversion speckle

The Shannon entropy $(S=-{\sum\nolimits_{i}}{p}_{i}{\log }_{2}({p}_{i}))$ is used to evaluate speckle entropy, where ${p}_{i}$ is the PDF of upconversion speckle image^52^. The statistical PDF of the upconversion speckle intensity distributions can be manipulated by varying the encoder area (see Supplementary Note 4), a process that is also characterized by changes in the speckle grain size. The grain size of the speckle is defined by the width of the autocorrelation function of the intensity speckle, which is well recognized and utilized in the characterization of speckle patterns^60^. To manipulate the encoder area, the distance between the illumination objective (Obj_1_) and the ground glass is modified, enabling the manipulation of the entropy of SWIR speckle based on the encoder area.

## Supplementary information

Supplementary Video 1 for number sequences

Supplementary Information

## Data Availability

The training datasets used are publicly available via Fashion-MNIST^61^, ImageNet^54^, and Ref. ^62^. Any additional data are available from the corresponding authors on request. Codes for the whole pipeline of this study is included in the link: https://github.com/withinWolfcontrol/S-ULRnet.
