# Supplementary material for: Upconversion optical entropy encoding for infrared complex-amplitude imaging
Source: Light Sci Appl. 2026 Mar 9;15:158. doi: 10.1038/s41377-026-02215-7 (PMC12968082; doi:10.1038/s41377-026-02215-7)
Supplement: Supplementary file 2 — Supplementary Information [file 41377_2026_2215_MOESM2_ESM.pdf]

Supplementary Information for
Upconversion optical entropy encoding for infrared complex-amplitude imaging

Sheng-ke Zhu[1†], Tuqiang Pan[2,3†], Chao-xian Tang[1†], Ai-Hua Li[4], Ze-huan Zheng[1], Yi Xu[2,3*], Xiangping Li[5*], Jin-hui Chen[1,6,7*]

[1] Institute of Electromagnetics and Acoustics, Key Laboratory of Electromagnetic Wave Science and Detection Technology, Xiamen University, Xiamen 361005, China.
[2] Key Laboratory of Photonic Technology for Integrated Sensing and Communication, Ministry of Education of China, Guangdong University of Technology, Guangzhou 510006, China.
[3] Institute of Advanced Photonics Technology, School of Information Engineering, Guangdong University of Technology, Guangzhou 510006, China.
[4] Department of Physics, Xiamen University, Xiamen 361005, China.
[5] Institute of Photonic Technology, College of Physics and Optoelectronic Engineering, Jinan University, Guangzhou 510632, China.
[6] Shenzhen Research Institute of Xiamen University, Shenzhen 518000, China.
[7] Innovation Laboratory for Sciences and Technologies of Energy Materials of Fujian Province (IKKEM), Xiamen 361005, China.
[†] These authors contributed equally to this work.
[*] Corresponding authors. Emails: yixu@gdut.edu.cn; xiangpingli@jnu.edu.cn; jimchen@xmu.edu.cn

Supplementary Notes

The supplementary notes are organized as follows. Supplementary Note 1 discusses performance comparisons among upconversion devices. Supplementary Note 2 provides characterizations of lanthanide transducers, including X-ray diffraction and upconversion spectral measurements. Supplementary Note 3 discusses the mapping relationship between visible speckles and near-infrared light fields. Supplementary Note 4 discusses the encoding mechanism of the scattering medium and the entropy analysis of the speckle image. Supplementary Note 5 describes the architectures of S-ULRnet. Supplementary Note 6 discusses the experimental setup for light field generation and measurement. Supplementary Note 7 discusses the dynamic range of modulation and power detection limit of the upconversion imaging system (UIS). Supplementary Note 8 discusses image evaluation metrics and training configurations of S-ULRnet. Supplementary Note 9 provides additional results of the retrieved biological organelles, irregular patterns, and speed sign images. Supplementary Note 10 discusses the configuration of Resnet50. Supplementary Note 11 provides the upconversion imaging results using a 980 nm laser and broadband infrared light excitation. Supplementary Note 12 discusses the long-term stability of the experimental setup.

Supplementary Note 1 — Performance comparisons among infrared upconversion devices.

Table S1 summarizes the imaging performance of the upconversion devices. Estimates of costs are based primarily on the functional materials and processing costs.

Table S1. Performance comparisons of infrared-to-visible upconversion devices in previous works.

Infrared detection methods /devices	Imaging dynamic range	Working wavelength	Power detection limit	Cost	Complex-amplitude imaging	Video rate
InGaAs camera (JCOPTIX AIC-NIR30GE)	8 bit	400-1700 nm	-	\$10000	×	✓
Parametric upconversion[1]	1 bit	2.9 µm (5 nm bandwidth)	-	∼ \$1000	×	-
Metalens[2]	1 bit	1105-1125 nm	1060 nW μm^{-2}	∼ \$100	×	×
Lanthanide upconversion[3]	1 bit	4.5–10.8 µm	0.3 nW μm^{-2}	-	×	×
Parametric upconversion[4]	1 bit	1064 nm	-	∼ \$1000	×	×
Nonlinear transmission matrices[5]	3 bit	800 nm	10^{12} nW μm^{-2}	-	✓	×
This work (Upconversion optical encoding)	8 bit	940-1020 nm & 1530-1565 nm	0.2 nW μm^{-2}	∼ \$50	✓	✓

Supplementary Note 2 — Characterizations of lanthanide transducers.

The fabrication process of the lanthanide transducer film is described in the Methods section of the main text. In Fig. S1a, the powder X-ray diffraction patterns confirm the host material of $ZnF_2(H_2O)_4$. Figure S1b displays the typical optical microscope image of spin-coating lanthanide transducer film in a glass substrate. Utilizing the large-area lanthanide transducer film, we verify its upconversion capabilities under structured short-wave-infrared (SWIR) light irradiation. When a SWIR laser beam (@1550 nm), shaped by the spatial light modulator (SLM), illuminates the lanthanide transducer film, a direct upconversion image is observed as shown in Fig. S1c. Note that a considerable noise background accompanies this direct upconversion image. When the lanthanide transducers are excited with 1550 nm radiation, the Er^{3+} ions absorb two photons successively and transition to a $^4I_{9/2}$ state. Subsequently, the excited Er^{3+} ions relax to $^4I_{11/2}$ state and then transfer energy to neighboring Yb^{3+} ions or Er^{3+} ions, promoting them to an excited state. Next, the excited Yb^{3+} or Er^{3+} ions transfer energy to another excited state of Er^{3+} ions, facilitating their transition to even higher energy states ($^4F_{9/2}$). Finally, the Er^{3+} ions at $^4F_{9/2}$ return to their ground state through radiative transition, emitting visible photons at approximately 660 nm. The inset of Fig. S1d shows the optical spectra of the upconversion transducers under 1550 nm excitation, including two major emission bands, corresponding to wavelengths of $\sim$ 660 nm and $\sim$ 980 nm, respectively. Figure S1d shows the photoluminescence intensity curve as a function of the power of a 1550 nm laser source. The strong nonlinear dependence on the power density for the upconversion photoluminescence may result in significant information loss in images. For example, Fig. S1e illustrates the direct infrared imaging results obtained via the lanthanide transducer film, revealing that detailed information is lost due to the threshold effect of upconversion.

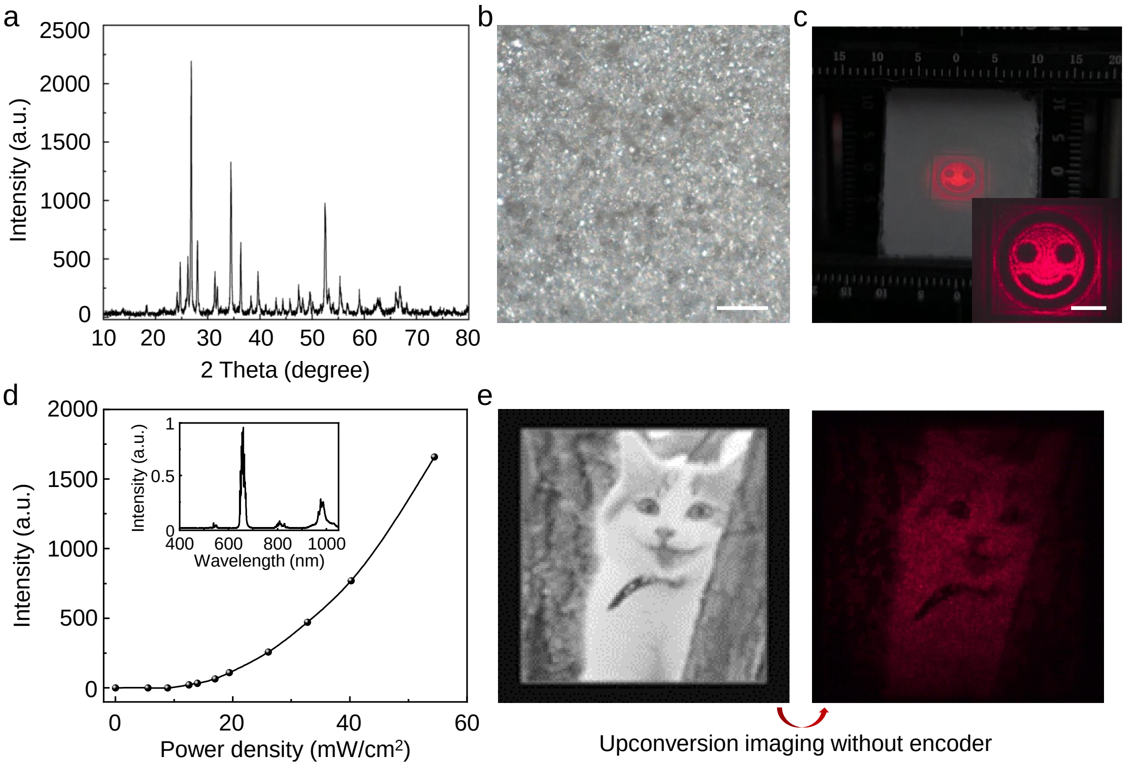

Figure S1. Characterizations of lanthanide transducer film. **a,** Powder X-ray diffraction pattern for $ZnF_2(H_2O)_4$:Yb^{3+}/Er^{3+} sample. **b,** Optical microscope image of $ZnF_2(H_2O)_4$:Yb^{3+}/Er^{3+} lanthanide transducer film. Scale bar: 30 μm. **c,** Photograph of the lanthanide transducer film under the illumination of SWIR light (@1550 nm). The illumination light is structured via a smiling face mask. The inset shows the enlarged view of upconversion images. Scale bar: 2 mm. **d,** The upconversion photoluminescence intensity of the lanthanide transducer film relating to the pump light power density (@ 1550 nm). The inset is the typical photoluminescence spectra under the excitation of a 1550 nm laser. **e,** The experimental upconversion imaging (right panel) under the illumination of a 1550 nm laser through an 8-bit photomask (left panel). The image information is partially lost due to the pump power threshold effect and nonlinear optics of lanthanide film. This image is adapted from the ImageNet dataset[6].

Supplementary Note 3 — Mapping relationship between visible speckles and incident SWIR light field.

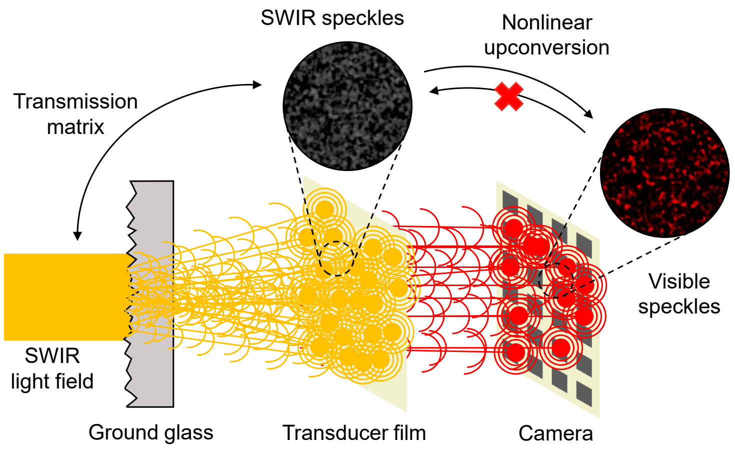

Figure S2. Mapping relationship between visible speckles and incident SWIR light-field. The SWIR light-field information cannot be retrieved by the conventional transmission matrix method using an upconversion speckle image because the nonlinear-optic process of upconversion destroys the linear mapping relationship based on the transmission matrix of ground glass.

Figure S2 shows a schematic of the SWIR light-field image system composed of the disordered encoder, lanthanide transducer film, and silicon-based image sensor (SIS). The complex-amplitude SWIR light field is denoted as a complex number that contains both amplitude and phase information, which can be expressed as:

$$\boldsymbol{E}_{\text{in}} = \hat{\boldsymbol{e}} \cdot A(x,y)e^{i\phi(x,y)} \tag{1}$$

where $A(x,y)$ and $\phi(x,y)$ represent the amplitude and phase distributions of the incident light field, respectively; $\hat{\boldsymbol{e}}$ indicates the unit vector of the electric field. Optical wave propagation through a highly scattering medium is a fundamental physical phenomenon relevant to numerous research areas, including imaging through turbid media[7] and information processing[8]. The relationship between the respective incident and scattered wave fields can be described by a transmission matrix (T)[9]:

$$\boldsymbol{E}_{\text{out}} = T \cdot \boldsymbol{E}_{\text{in}} \tag{2}$$

Then, we select lanthanide-doped luminescent materials as upconversion transducers, which can efficiently convert SWIR light into visible light detectable by a conventional SIS. The mapping relationship between the visible speckles and incident SWIR light field can be described by a nonlinear transfer function as follows:

$$I = F(|\boldsymbol{E}_{\text{out}}|^2) = F(|T \cdot \boldsymbol{E}_{\text{in}}|^2) \tag{3}$$

where $F(\cdot)$ indicates the nonlinear operation for the upconversion process and correlates the SWIR speckle field with the converted visible speckles (I). For example, the SWIR speckles can be mapped to visible speckles based on the measured nonlinear upconversion relationship, as shown in Fig. S1d. Thus, the output visible speckle intensity recorded by an SIS can be expressed as follows:

$$I = H(\boldsymbol{E}_{\text{in}}) \tag{4}$$

where $H(\cdot)$ represents the mapping relationship between the incident SWIR light field ($\boldsymbol{E}_{\text{in}}$) and the intensity distribution of the visible speckles. Therefore, the input SWIR light field can be formally retrieved by conducting an inverse mapping operation as:

$$\boldsymbol{E}_{\text{in}} = H^{-1}(I) \tag{5}$$

where H^{-1} is the inverse mapping of the Supplementary Eq. 4. The nonlinear conversion process of SWIR light to visible light destroys the linear mapping relationship of the transmission matrix. Besides, the limited efficiency of the luminescent materials used and the photosensitivity of SIS lead to the loss of detailed information of input SWIR field and add to the difficulty of perfect upconversion imaging[10]. Crucially, no physics-based theory has been reported so far that can effectively solve the problem of image retrieved under such strong nonlinear relations. We overcome this limitation and retrieve H^{-1} via data-driven deep learning methods[11], as discussed in the main text.

Supplementary Note 4 — Encoding mechanism via scattering medium and speckle image entropy analysis.

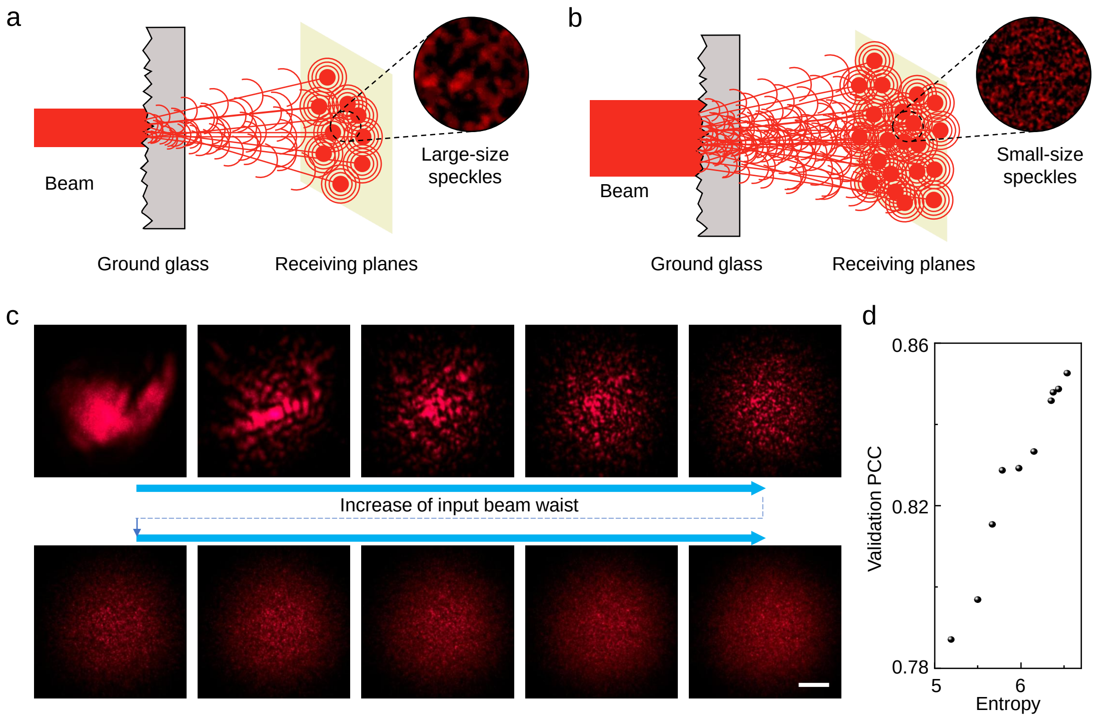

Figure S3. The effects of input beam waist on the performance of SWIR light-field sensor. a, Schematic illustration of large-size speckle spots produced by ground glass when the illumination light source is of a small beam waist. **b,** Schematic illustration of small-size speckle spots produced by ground glass when the illumination light source is of large beam waist. **c,** Experimental upconversion speckle images with different input beam waists. Scale bar: 500 µm. **d,** Validation PCC of retrieved images on speckle entropy. Data are presented as mean values processed from 5000 measurements.

This section begins with an intuitive method for deducing the communication degrees of freedom limit between two finite two-dimensional planes with arbitrary source distributions under paraxial approximation[9,12]. For the incident and receiver planes with areas A_S and A_R, respectively, the minimum spot (a) generated by incident plane A_S on receiver plane A_R can be obtained from the concept of solid angle as:

$$a \sim \frac{\lambda^2 D^2}{A_S} \tag{6}$$

where λ is the free-space wavelength, D is the distance between the two planes. As each spot can be utilized for independently coding information, the information capacity can be intuitively regarded as the maximum containable number of spots on A_R:

$$N \sim \frac{A_R}{a} \tag{7}$$

which is:

$$N \sim \frac{A_S A_R}{\lambda^2 D^2} \tag{8}$$

Hence, by incrementally enhancing the waist of the input beam, the diffraction-limited spot size (a) of the speckle pattern is decreased steadily since the physical size (A_S) of the incident aperture is increased (Fig. S3a- b), enabling the speckle to carry more information. Figure S3c shows the experimental upconversion speckle images captured with varying input beam waists, clearly demonstrating a correspondence between the experimental outcomes and the predictions of the theoretical model. Since the input SWIR light field is mapped onto a speckle pattern, the information capacity of the latter is crucial in determining the performance of the proposed sensor when the information is retrieved by a deep neural network. Image entropy is a statistical

metric of randomness, serves as a valuable tool in characterizing the information encapsulated within the speckle image. To access the speckle entropy, we employ Shannon entropy, defined as follows:

$$S = -\sum_i p_i \log_2 (p_i) \tag{9}$$

where p_i is the probability density function (PDF) of the upconversion speckle image. Figure S3d shows the comparison of validation PCC of phase image retrieving derived from different entropy resulting from varying sizes of input beam waists.

Supplementary Note 5 — Architectures of the S-ULRnet.

Figure 3a in the main text shows the architecture of our designed S-ULRnet. The detailed structure of U-CNN is shown in Figure S4. Table S2 displays the number of convolution kernels and the stride for each convolutional layer in the U-CNN architecture. The input and output sizes for each block are also provided. The stride (S_1, S_2) for the convolutional layer in the last Res-Encoder (block number 4) is set to 1, ensuring that the output is not further downsampled. The first Res-Decoder (block number 5) does not require a transposed convolutional layer because it does not need to upsample the output from the previous block. Instead, it performs channel concatenation directly with the output from the skip connection.

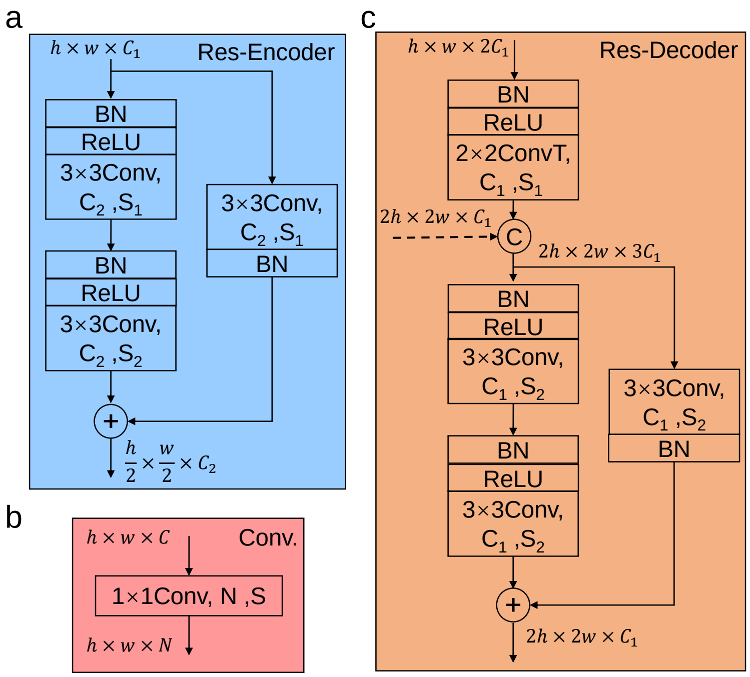

Figure S4. Specific configuration of each module within U-CNN. a, The implementation of Res-Encoder, where there are two convolutional layers in the main path and one convolution on the branch path. The output size of the two paths is the same, and the output is formed by element-wise addition. **b,** The Conv. channel matching is implemented with a convolutional layer using a 1×1 convolution kernel and a stride of 1. **c,** The Res-Decoder implementation has two inputs: the output from the previous module and the skip connection from the cross-layer. The feature map from the previous module is first upsampled using a transposed convolutional layer, then concatenated with the skip connection output from the cross-layer. The resulting feature map is then compressed using a structure similar to that of the Res-Encoder.

Table S2. The configuration of parameters within the convolutional block. "C" represents the number of convolution kernels, indicating the number of channels in the feature map generated by the convolution layer. "S" represents the stride of the convolution operation.

Block number	C_1	C_2	S_1	S_2	Input size	Output size
1	1	64	1	1	92× 92× 1	92× 92× 64
2	64	128	2	1	92× 92× 64	46× 46× 128
3	128	256	2	1	46× 46× 128	23× 23× 256
4	256	512	1	1	23× 23× 256	23× 23× 512
5	256	N/A	N/A	1	23× 23× 512<br>23× 23× 256	23× 23× 256
6	128	N/A	2	1	23× 23× 256<br>46× 46× 128	46× 46× 128
7	64	N/A	2	1	46× 46× 128<br>92× 92× 64	92× 92× 64

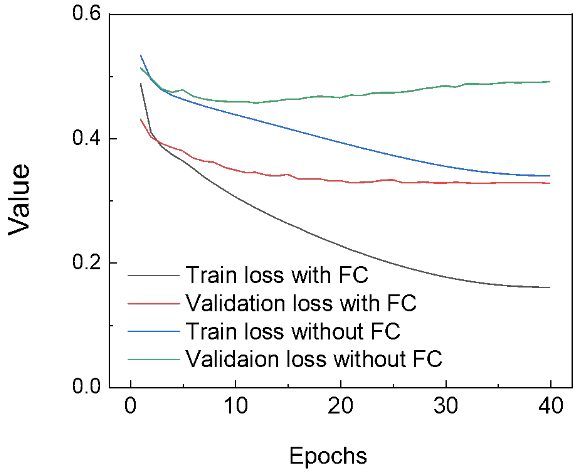

Figure S5. Ablation study on the fully connected (FC) layer. The plot shows the training and validation loss curves (SSIM values) over 40 epochs for S-ULRnet. The removal of the FC layer leads to a substantial increase in both training and validation loss, confirming its necessity for the model's effectiveness.

Supplementary Note 6 — Experimental setup.

The experimental setup is shown in Fig. S6. The details of experimental parameters can be found in the Methods section of the main text.

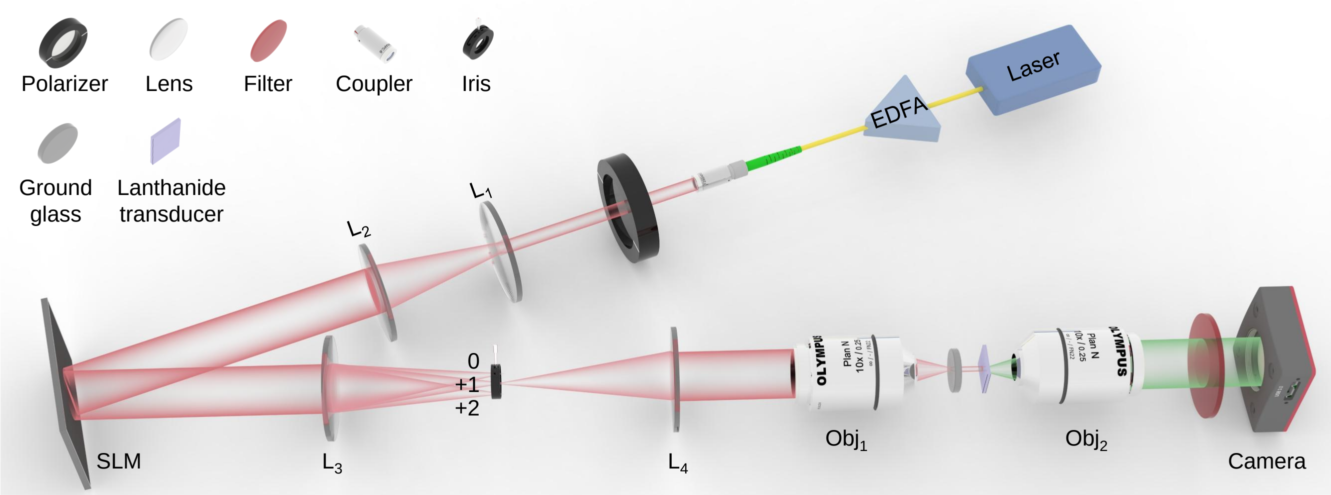

Figure S6. Experimental setup for structured SWIR light-field generation and upconversion detection. The structured light field is created using a phase-only spatial light modulator (SLM). The apparatus for the SWIR light-field sensor is composed of ground glass, upconversion transducer film, and SIS.

Table S3. The detailed parameters of operation time. The upload time of hologram can be minimized by using a digital mirror device (Fldiscovery, F4320 DDR 0.95 1080P), which is substantially smaller than 0.05 ms. Therefore, the hologram uploading time can be excluded for consideration.

Step of single-shot process	Specific operation	Time consumption	Device/software dependence
1. Hologram upload	Loading the pre-calculated phase hologram onto SLM	$\sim$ 16.7 ms	Limited by the SLM's hardware refresh rate
2. Image capture	Acquiring the diffraction pattern of the object using a CMOS camera	$\sim$ 35 ms	Determined by the camera's photosensitivity and gain
3. Complex field retrieval	Network inference (input: captured speckle pattern; output: reconstructed complex field)	$\sim$ 5 ms	Dependent on the GPU (Nvidia RTX4090 graphics card) and optimized network structure
Total time tested		$\sim$ 56.7 ms	
Effective reconstruction time (excluding hologram upload)	Steps 2+3	$\sim$ 40 ms	

Supplementary Note 7 — The dynamic range of modulation and power detection limit of upconversion image sensor.

Figures S7a and S7b show the retrieved results of complex-amplitude SWIR light field with 2-bit and 8-bit dynamic range of modulation, respectively. Based on the typical recovery results and statistical outcomes presented (Figure S7c), we claim the developed UIS can achieve excellent imaging performance for 8-bit grayscale of both amplitude and phase.

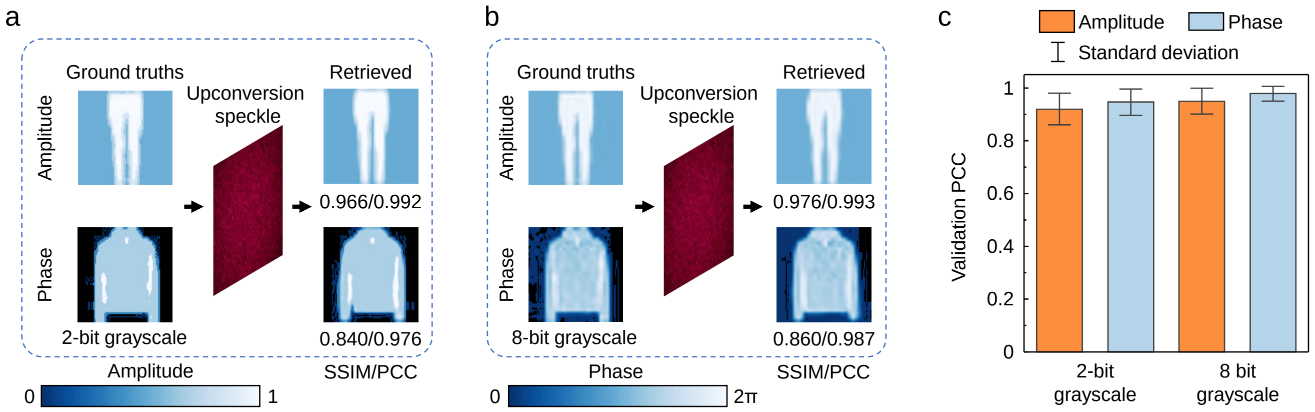

Figure S7. Upconversion imaging performance with different dynamic range digital image. a,b The ground truths, the single-shot upconversion speckle, and the corresponding retrieved light-field information by the S-ULRnet are shown using the 2-bit grayscale images (**a**) and 8-bit grayscale images (**b**). **c,** Validation PCC of the complex-amplitude image, with 2-bit grayscale images and 8-bit grayscale images. The validation PCC is averaged over 3000 measurements from the Fashion-MNIST[13]. The error bar indicates the standard deviation of retrieved fidelity.

To characterize the infrared response of the UIS, the integration time used for the SIS is 35 ms. The power detection limit is calculated using the following equation: power (density) detection limit=(Minimum detection power)/(effective area), in which the minimum detection power is recorded when the exposure time of the SIS reaches 35 ms and the retrieved accuracy is good. The set exposure time of 35 ms and inference time of 5 ms achieves a video frame rate. Figure S8 illustrates the retrieved results under different power densities. It is observed that when the power density reaches 0.2 nW μm^{-2}, the intensity of upconversion speckle is sufficient to reconstruct target images with acceptable quality, while simultaneously allowing the sensor's acquisition speed to reach video frame rates. This means the sensor's response time is sufficiently fast to capture images for smooth video playback at this power density level.

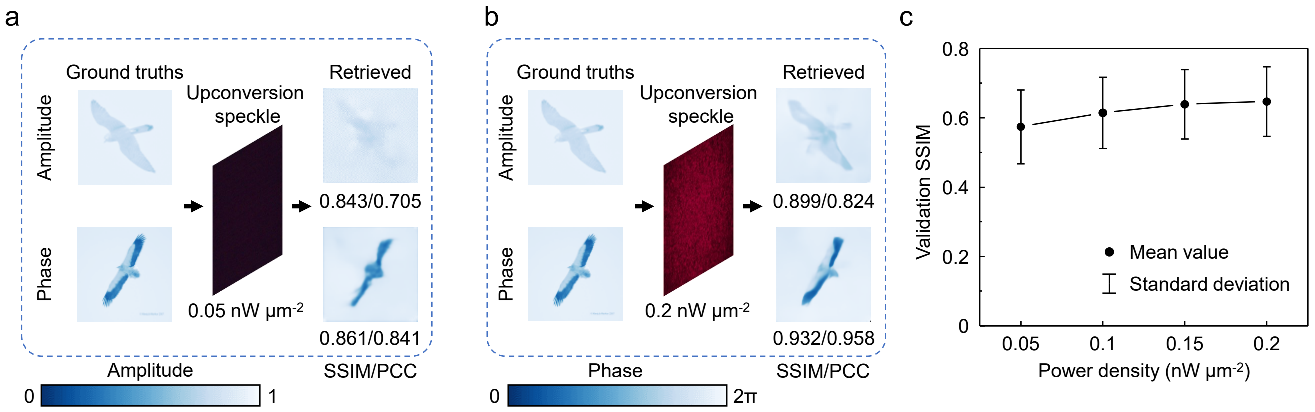

Figure S8. Upconversion imaging performance with different power density. a,b The ground truths, the single-shot upconversion speckle, and the corresponding retrieved light-field information by the S-ULRnet are shown using the power density of 0.05 nW/μm^2 (**a**) and power density of 0.2 nW/μm^2 (**b**). **c,** Validation SSIM of the complex-amplitude image with different power densities. The validation SSIM is averaged over 5000 measurements from the ImageNet dataset[14]. The error bar indicates the standard deviation of retrieved fidelity. The integration time for the SIS is set as 35 ms.

Supplementary Note 8 — Image evaluation metrics and training configuration of S-ULRnet.

To evaluate the quality of the retrieved images, we introduce the Pearson correlation coefficient (PCC)[15] and structural similarity index (SSIM)[14]. The PCC is defined as:

$$\mathrm{PCC}(G,R) = \frac{\sum_i \left(g_i - \bar{G}\right)\left(r_i - \bar{R}\right)}{\sqrt{\sum_i \left(g_i - \bar{G}\right)^2 \sum_i \left(r_i - \bar{R}\right)^2}} \tag{10}$$

where g_i and r_i represent the pixelated values (pixel index i) of the ground truth and retrieved images, respectively. $\bar{G}$ and $\bar{R}$ are the mean values of g and r, respectively. SSIM evaluates image quality based on three aspects: brightness, contrast, and structure, which align better with the visual effects perceived by human vision. The calculation formula is as follows:

$$\mathrm{SSIM}(G,R) = \frac{(2\mu_g \mu_r + C_1)(2w_{gr} + C_2)}{\left(\mu_g^2 + \mu_r^2 + C_1\right)\left(w_g^2 + w_r^2 + C_2\right)} \tag{11}$$

where μ_g and μ_r are the average of the ground truth and retrieved images, respectively; w_g and w_r are the standard deviations of the ground truth and retrieved images, respectively; w_{gr} is the cross-covariance between the ground truth and retrieved images; C_1 and C_2 are regularization parameters.

The S-ULRnet is implemented using Python 3.9.13 in PyTorch 2.0.0. The model is trained using the AdamW optimizer, which is an improved version of the Adam optimizer known for better generalization performance[16]. The initial learning rate is set at 1×10^{-6} and increases to 1×10^{-3} after 5 epochs of warm-up. The learning rate decreases to 1×10^{-9} in the final epoch, following a cosine annealing schedule. Throughout the training process, the loss function value does not show significant fluctuation[17]. The total number of training epochs is set to 40 to ensure training convergence. SSIM is chosen as the loss function, and the specific implementation details can be found in Ref.[18,19]. Figure S9 shows averaged loss functions, SSIMs, and PCCs of the training dataset and validation dataset during the training procedure. Figure S10 shows the PCC distribution of the validation dataset. The retrieved PCC utilizing the same training configuration of S-ULRnet converges to 0.99 after training, indicating that high fidelity of the retrieved image information has been achieved. Table S4 summarizes of performance of the retrieved image performance for different modulation schemes and the used image datasets, i.e., Fashion-MNIST and ImageNet. For the image reconstruction task of speed limit signs, due to the small size of the dataset in Ref.[20], we mix them with the ImageNet dataset and train them together.

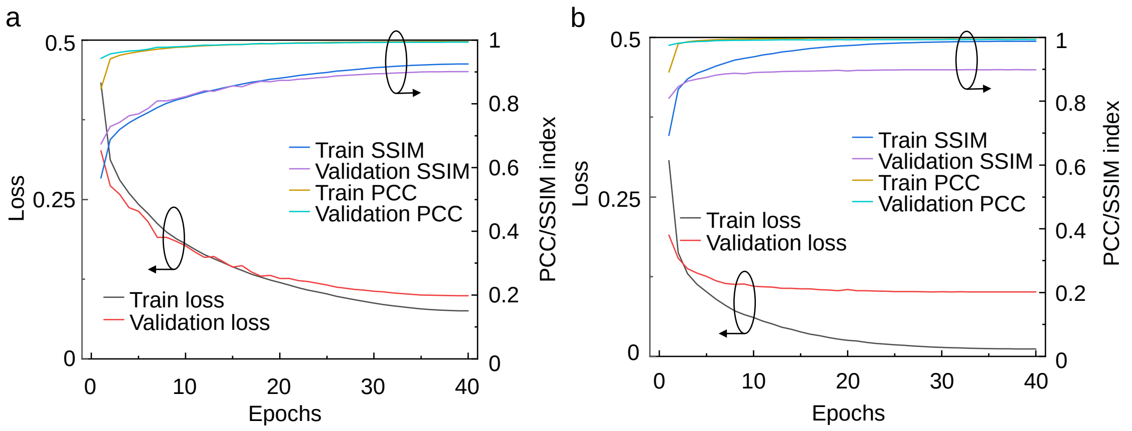

Figure S9. Average loss functions, SSIMs, and PCCs of the training dataset and validation dataset during training procedures. a, Training process of retrieved amplitude-based images. **b,** Training process of retrieved phase-based images. Here, the Fashion-MNIST dataset is used in amplitude/phase image modulation[13].

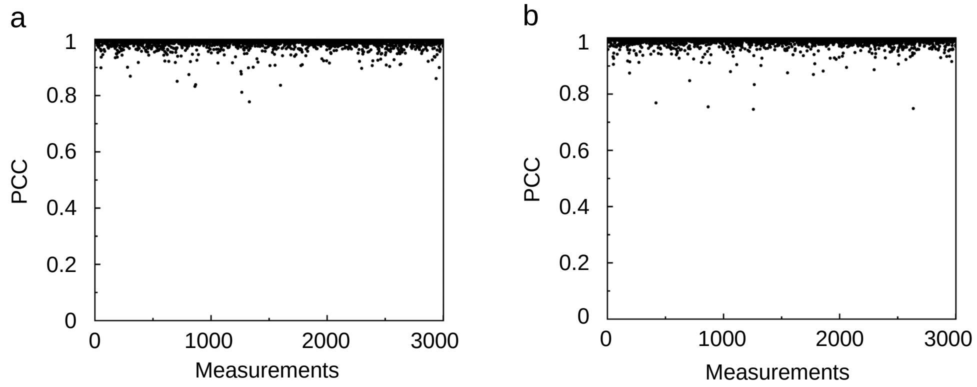

Figure S10. PCC distribution of the validation dataset. a, Validation PCC of retrieved amplitude-based images. **b,** Validation PCC of retrieved phase-based images. Here, the Fashion-MNIST dataset is used for amplitude/phase image modulation[13].

Table S4. Summaries of performance of image retrieval for different modulation schemes and image datasets. Dataset type, modulation scheme, dataset size, and averaged fidelity (SSIM and PCC) are labeled. The resolution of all images is 92×92. All validation sets are 10% of their corresponding datasets.

Dataset	Modulation scheme	Dataset size	Average fidelity (SSIM/PCC)
Fashion-MNIST	Amplitude	30000	0.901/0.989
Fashion-MNIST	Phase	30000	0.899/0.989
Fashion-MNIST	Complex amplitude	30000	0.884/0.974
ImageNet	Amplitude	50000	0.773/0.917
ImageNet	Phase	50000	0.712/0.853
ImageNet	Complex amplitude	50000	0.674/0.741
ImageNet & speed limit sign image from Ref.[20]	Amplitude	51712	0.932/0.954 (for speed limit signs)

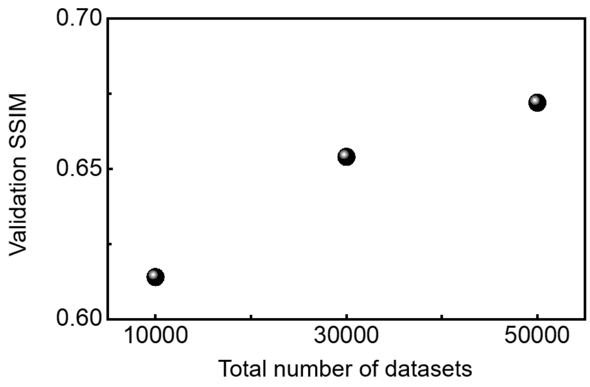

Figure S11. The effect of dataset size on the reconstruction performance, using the ImageNet dataset with complex amplitude modulation. The performance is evaluated using the SSIM on the validation set, which was consistently held at 10% of the total dataset size. Increasing the data amount for training the neural network can improve the performance of S-ULRnet.

Supplementary Note 9 —Biological organelles, irregular patterns and speed sign images retrieved.

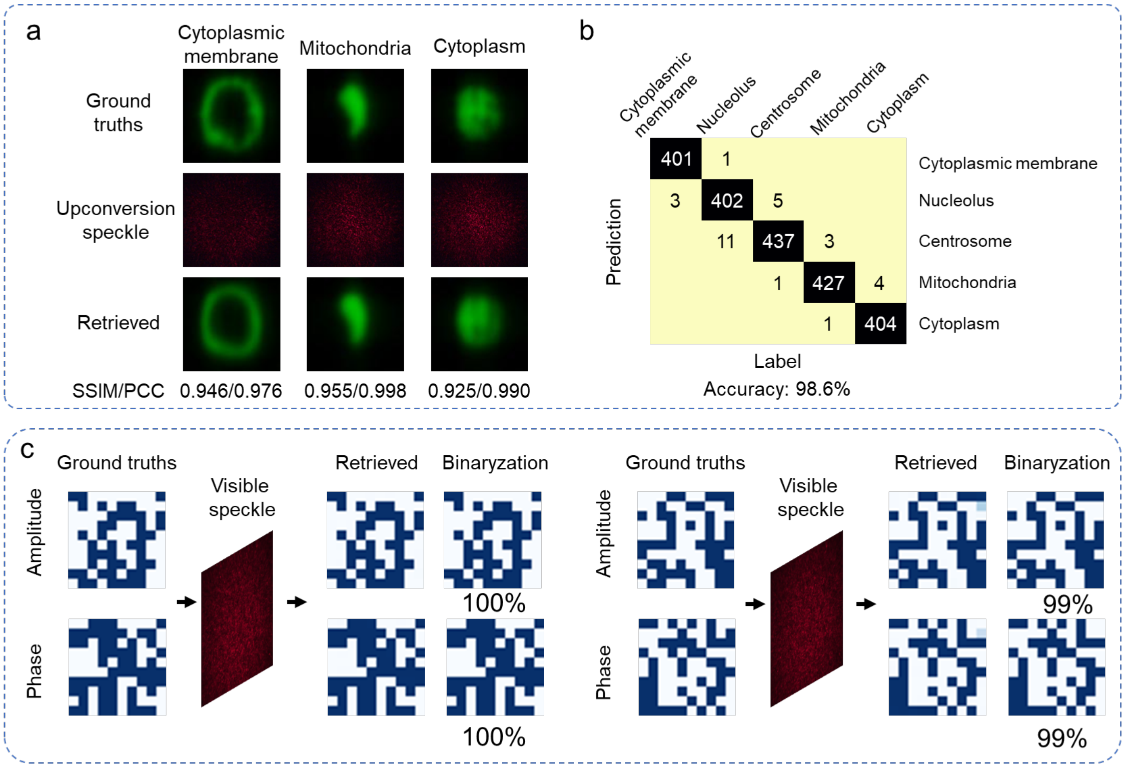

Figure S12. a, Retrieved results of cell organelle images. The ground truths, the single-shot upconversion speckles, and the corresponding retrieved amplitude information by the S-ULRnet are shown. The corresponding SSIM and PCC of the retrieved results are labeled, respectively. **b,** The results of classifying cell organelles. The used images (ground truths) in (a) are adapted from Ref[20]. **c,** Uncorrelated binary information is used to mimic the irregular patterns. The ground truths, the single-shot upconversion speckles, and the corresponding retrieved amplitude and phase information by the S-ULRnet are shown.

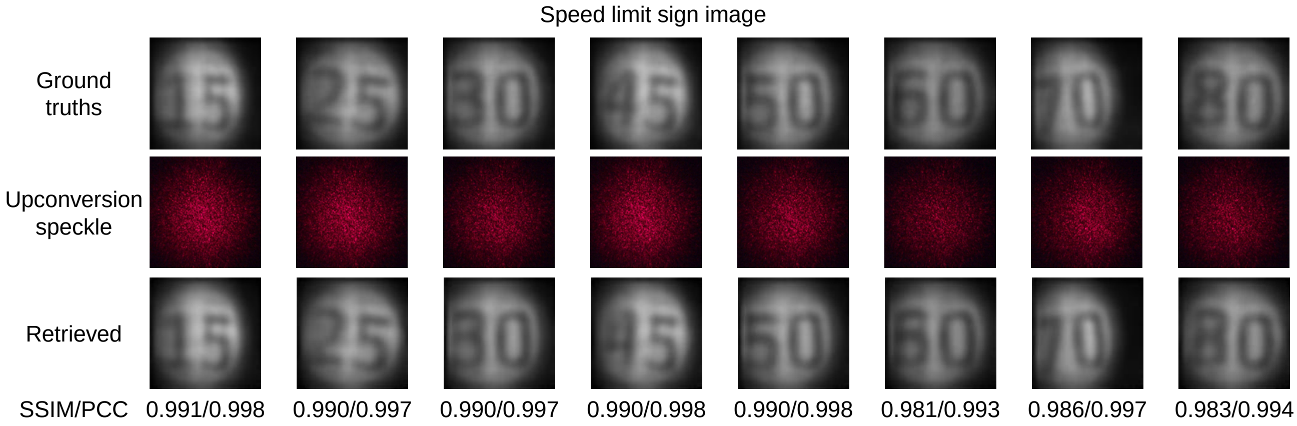

Figure S13. Retrieved results of traffic sign images. The ground truths, the single-shot visible speckle output and the corresponding retrieved amplitude information by the S-ULRnet are shown. The corresponding SSIM and PCC of the retrieved results are labeled, respectively.

Supplementary Note 10 — Detailed configuration of Resnet50.

The dataset used for the classification task (Resnet50, see Fig. S14) is expanded by rotating the images used in Ref.[20]. For the speed limit signs classification task, the total dataset size is 6720, which is also from the dataset provided in Ref.[20]. For the classification task, labeled datasets are randomly split into training, validation, and testing sets under the ratio of 8:1:1. The neural network model is implemented using Python 3.9.13 in PyTorch 2.0.0. The workstation used to train and test the neural network is equipped with an Nvidia RTX4090 graphics card.

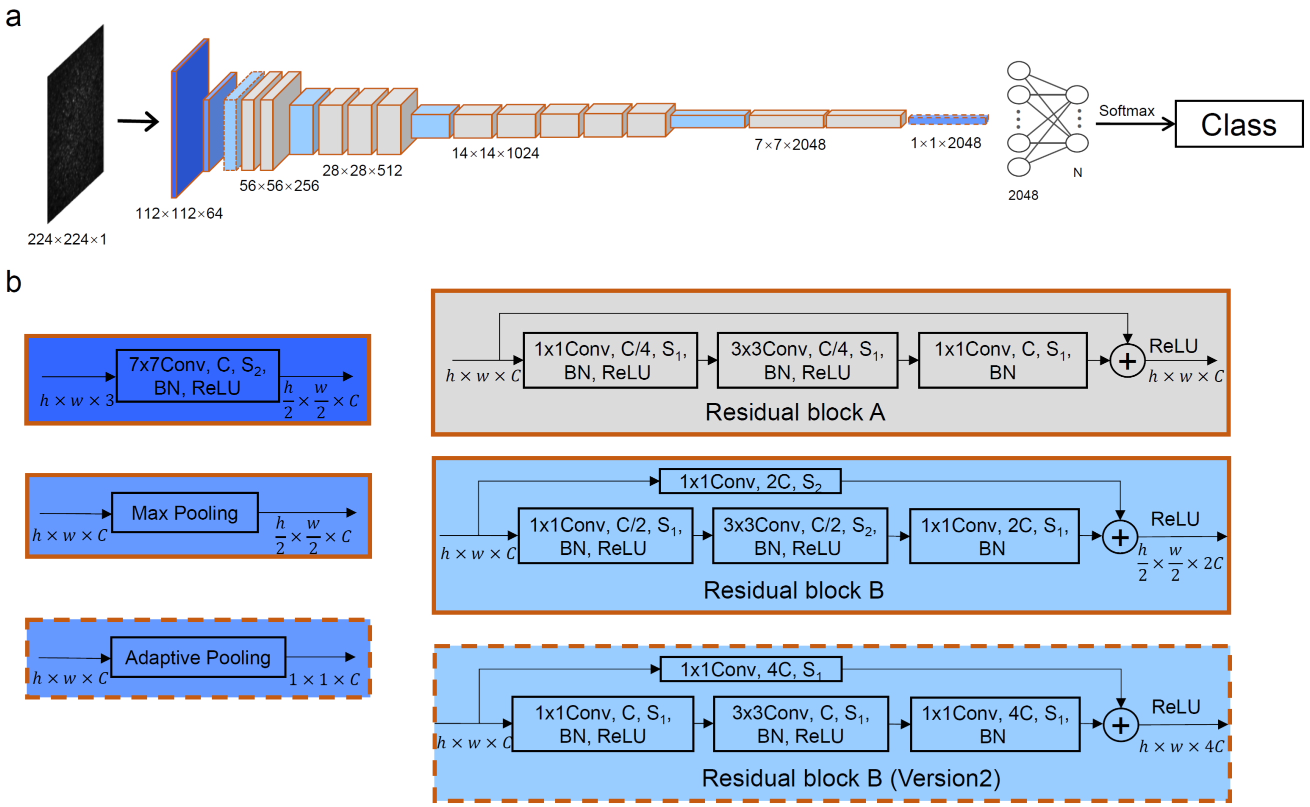

Figure S14. Standard Resnet50 network structure for upconversion image classification. a, Standard Resnet50 architecture. The red-channel image of the upconversion speckle is used as the input, and the convolutional layer and pooling layer are used for downsampling and feature extraction. Finally, a fully connected layer is used as the output layer, and the softmax function is used to convert the probability distribution of a limited number of specified categories. **b,** Specific configuration of each residual convolutional block.

Supplementary Note 11 — Complex-amplitude image retrieval under a 980 nm laser and broadband infrared light excitation.

Since the energy level between $^2F_{5/2}$ and $^2F_{7/2}$ of Yb^{3+} ion accords to the 980 nm pump photon, the lanthanide transducers with Yb^{3+} ions also emit visible red light under the illumination of a 980 nm laser. Figure S15 shows exemplary visualization of retrieved complex-amplitude images under the excitation of a 980 nm laser. Both amplitude and phase information encoded in the upconversion speckles can be retrieved with high fidelity using the proposed UIS. The average SSIM/PCC of the retrieval light field is 0.849/0.948 for fashion images and 0.659/0.704 for general natural scene images.

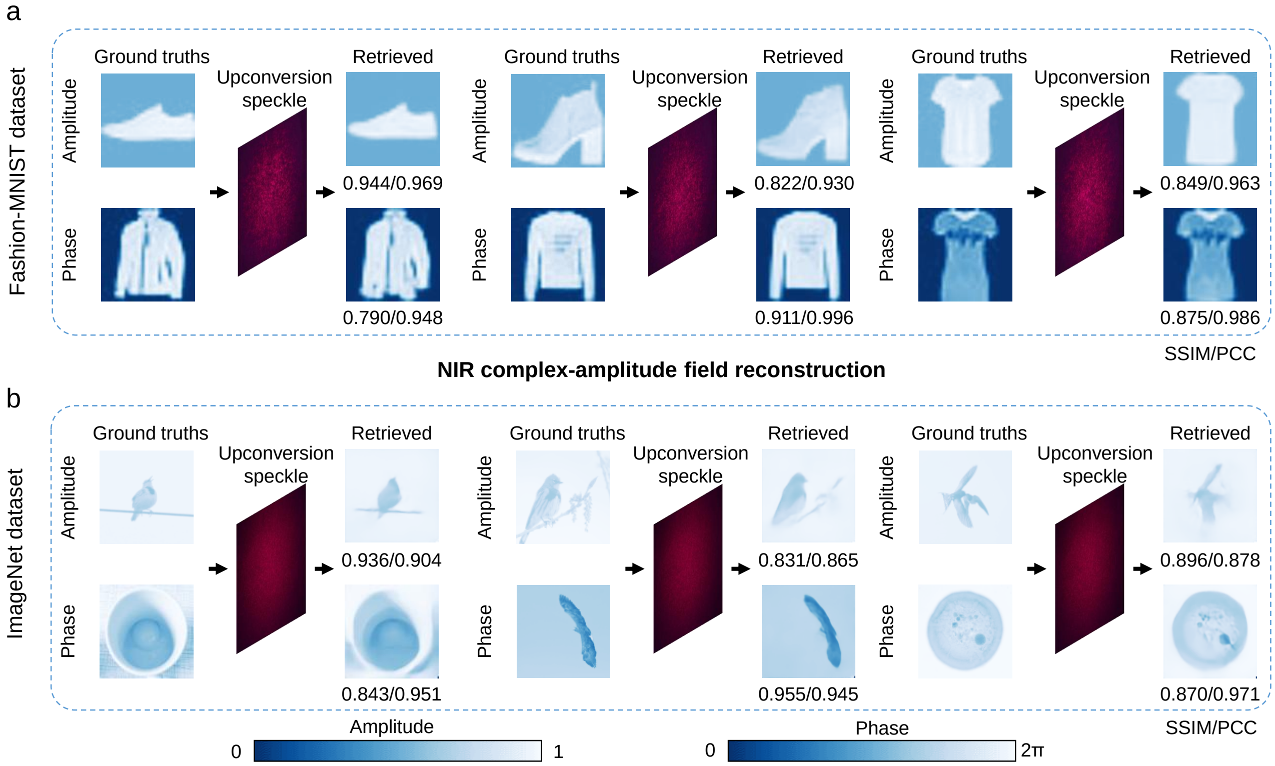

Figure S15. Complex-amplitude image retrieved under the excitation of a 980 nm laser. The ground truths, the visible speckle, and the corresponding retrieved light-field information by the S-ULRnet are shown, where their corresponding SSIM and PCC are given. These images in **(a)** are adopted from the Fashion-MNIST dataset[13] and the images in **(b)** are adopted from the ImageNet dataset[6].

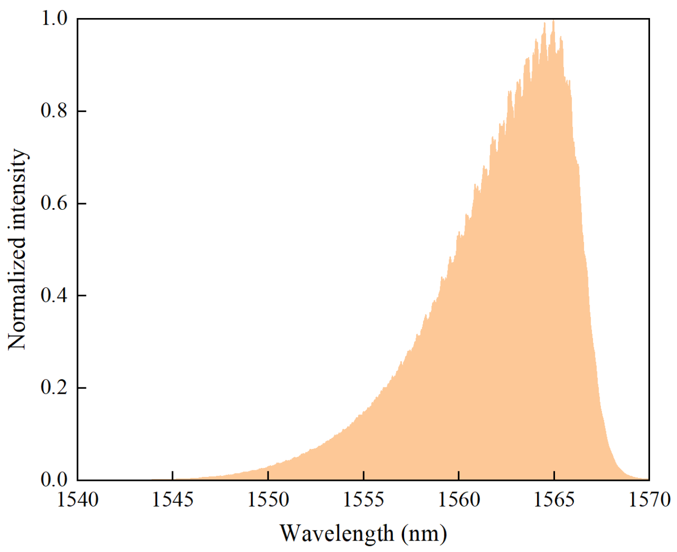

Figure S16. **The optical spectrum of a broadband infrared light source.**

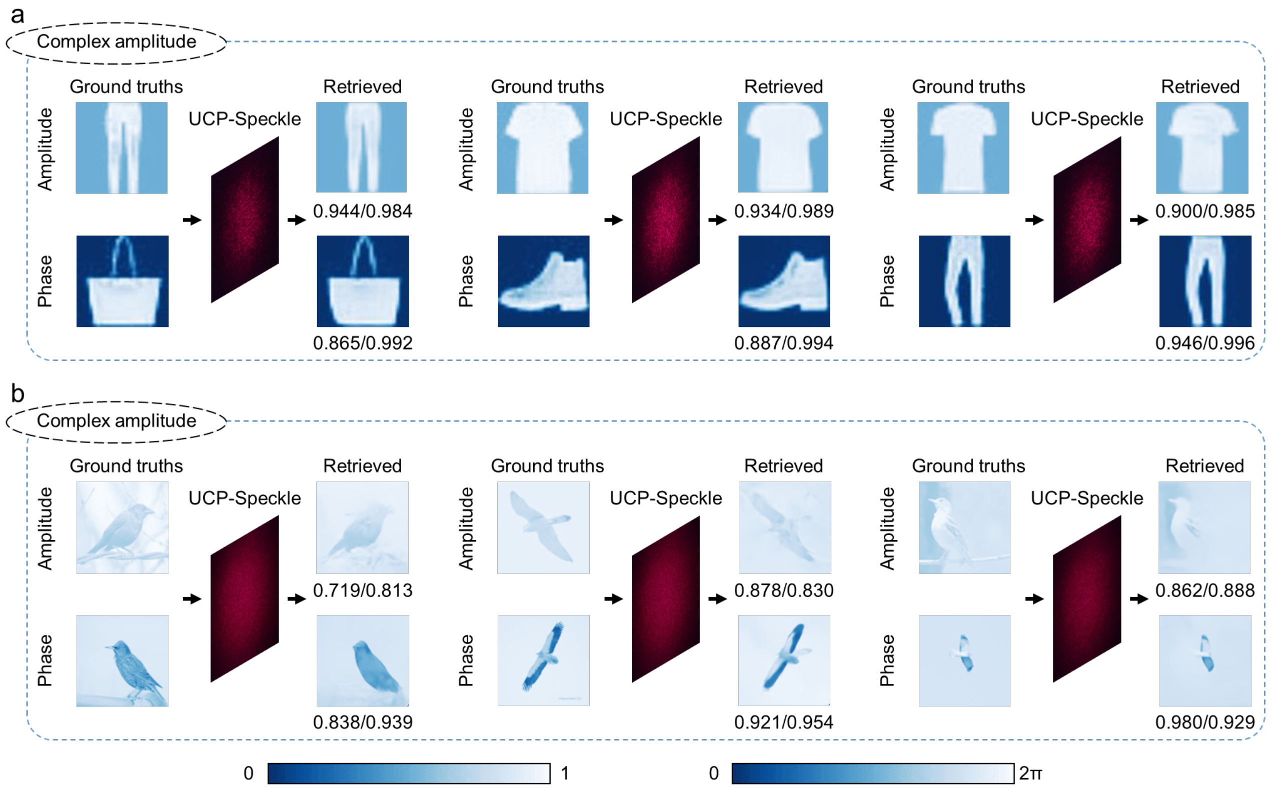

Figure S17. **Experimental validation of the method's robustness and generalization using a broadband light source.** Representative reconstruction results are shown for (a) the Fashion-MNIST dataset and (b) the ImageNet dataset.

Supplementary Note 12 — Time-domain stability monitoring of SWIR upconversion imaging.

To monitor the time-domain stability of the SWIR UIS, the PCC evaluating the correlation between an instantaneous speckle pattern (every 15 minutes) with the first one is calculated, as shown in Figure S18. The proposed UIS can maintain high repeatability (PCC > 0.96) after 24 hours, which is crucial for long-term applications.

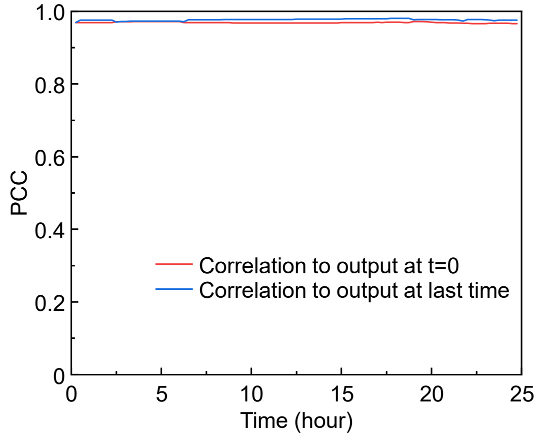

Figure S18. Time-domain stability monitoring of SWIR UIS under a fixed input light field. The PCC evaluating the correlation between an instantaneous speckle pattern (every 15 minutes) with the first one is presented.

References

1. Dam, J. S., Tidemand-Lichtenberg, P. & Pedersen, C. Room-temperature mid-infrared single-photon spectral imaging. *Nat. Photonics* **6**, 788–793, DOI: https://doi.org/10.1038/nphoton.2012.231 (2012).
2. Schlickriede, C. *et al.* Imaging through nonlinear metalens using second harmonic generation. *Adv. Mater.* **30**, 1703843, DOI: https://doi.org/10.1002/adma.201703843 (2018).
3. Liang, L., Wang, C., Chen, J., Wang, Q. J. & Liu, X. Incoherent broadband mid-infrared detection with lanthanide nanotransducers. *Nat. Photonics* **16**, 712–717, DOI: https://doi.org/10.1038/s41566-022-01042-7 (2022).
4. Qiu, X., Li, F., Zhang, W., Zhu, Z. & Chen, L. Spiral phase contrast imaging in nonlinear optics: seeing phase objects using invisible illumination. *Optica* **5**, 208–212 (2018).
5. Moon, J., Cho, Y.-C., Kang, S., Jang, M. & Choi, W. Measuring the scattering tensor of a disordered nonlinear medium. *Nat. Phys.* **19**, 1709–1718 (2023).
6. Deng, J. *et al.* Imagenet: A large-scale hierarchical image database. In *2009 IEEE Conference on Computer Vision and Pattern Recognition*, 248–255, DOI: https://doi.org/10.1109/CVPR.2009.5206848 (2009).
7. Popoff, S., Lerosey, G., Fink, M., Boccara, A. C. & Gigan, S. Image transmission through an opaque material. *Nat. Commun.* **1**, 81, DOI: https://doi.org/10.1038/ncomms1078 (2010).
8. Ott, J. R., Mortensen, N. A. & Lodahl, P. Quantum interference and entanglement induced by multiple scattering of light. *Phys. Rev. Lett.* **105**, 090501, DOI: https://doi.org/10.1103/PhysRevLett.105.090501 (2010).
9. Yu, H. *et al.* Measuring large optical transmission matrices of disordered media. *Phys. Rev. Lett.* **111**, 153902, DOI: https://doi.org/10.1103/PhysRevLett.111.153902 (2013).
10. Yi, L., Tan, H. Q., Hou, B. & Liu, X. X-ray-to-NIR multi-wavelength imaging through stochastic photoluminescence and compressed encoding. *Matter* **7**, 2431–2447, DOI: https://doi.org/10.1016/j.matt.2024.02.014 (2024).
11. Yuan, S. *et al.* Geometric deep optical sensing. *Science* **379**, eade1220, DOI: https://doi.org/10.1126/science.ade1220 (2023).
12. Miller, D. A. B. Communicating with waves between volumes: evaluating orthogonal spatial channels and limits on coupling strengths. *Appl. Opt.* **39**, 1681–1699, DOI: https://doi.org/10.1364/AO.39.001681 (2000).
13. Xiao, H., Rasul, K. & Vollgraf, R. Fashion-mnist: a novel image dataset for benchmarking machine learning algorithms. *arXiv preprint arXiv:1708.07747* DOI: https://doi.org/10.48550/arXiv.1708.07747 (2017).
14. Wang, Z., Bovik, A., Sheikh, H. & Simoncelli, E. Image quality assessment: from error visibility to structural similarity. *IEEE Transactions on Image Process.* **13**, 600–612, DOI: https://doi.org/10.1109/TIP.2003.819861 (2004).
15. Sedgwick, P. Pearson's correlation coefficient. *BMJ* **345**, e4483, DOI: https://doi.org/10.1136/bmj.e4483 (2012).
16. Loshchilov, I. & Hutter, F. Decoupled weight decay regularization. *arXiv preprint arXiv:1711.05101* DOI: https://doi.org/10.48550/arXiv.1711.05101 (2017).
17. Liu, Z. *et al.* A convnet for the 2020s. In *Proceedings of the IEEE/CVF conference on computer vision and pattern recognition*, 11976–11986 (2022).
18. Kastryulin, S., Zakirov, D. & Prokopenko, D. PyTorch Image Quality: Metrics and measure for image quality assessment (2019). Open-source software available at https://github.com/photosynthesis-team/piq.
19. Kastryulin, S., Zakirov, J., Prokopenko, D. & Dylov, D. V. Pytorch image quality: Metrics for image quality assessment, DOI: 10.48550/ARXIV.2208.14818 (2022).
20. Wang, T. *et al.* Image sensing with multilayer nonlinear optical neural networks. *Nat. Photonics* **17**, 408–415, DOI: https://doi.org/10.1038/s41566-023-01170-8 (2023).
